# Requirements for set-off in Japanese law

**DOI:** 10.12688/f1000research.161862.1

**Published:** 2025-03-03

**Authors:** Hiroki OKAMOTO

**Affiliations:** 1 University of Tsukuba, Ibaraki, Japan

**Keywords:** Japanese Civil Code, Japanese Bankruptcy Act, Set-off, Requirements for Set-off

## Abstract

As is already known, the Japanese Civil Code (JCC), enacted in 1896, was formulated with reference to the discipline of many foreign laws, including the French Civil Code (FCC) and the draft of the German Civil Code (BGB) at the time. Among the objects of discipline stipulated in the JCC, the concept of German law was widely adopted for the provisions on set-off. However, in the interpretation of these provisions, the Japanese Supreme Court sometimes adopted views that differed from the provisions of BGB and its interpretation. This case law was clarified when the Law of Obligations of the JCC was revised in 2017. This paper explains the various set-off requirements stipulated by Japanese current law.

In Japan, the conditions under which the statutory requirements for set-off are satisfied are described as “eligibility for set-off” (Sousai-Tekijou), and the legal status of the parties in the set-off is called the right to set-off. Even if this eligibility for set-off arises, the parties may not exercise the right to set-off if there are circumstances that restrict the set-off. In addition, the Japanese Bankruptcy Act (JBA) modifies the requirements provided by the JCC to accommodate bankruptcy proceedings.

In this paper, we will review in order the affirmative requirements for creating eligibility for set-off, the negative requirements for restricting the exercise of the right to set-off, and the requirements for set-off in bankruptcy proceedings. Please note that under Japanese law, when A intends to extinguish B’s claim β against him by set-off against his claim α, A’s claim α is referred to as an active claim (Jido-Saiken, Aktivforderung), and B’s cross-claim β as a passive claim (Judo-Saiken, Passivforderung). This paper will also be described based on this terminology ^1^.

## 1. Affirmative requirements

### 1-1 Reciprocity of obligations

1-1-1 *Mutual debts between the two parties*

The most basic requirement for set-off is that two persons are mutually responsible for the debts (JCC Art. 505 (1)). The law provides for the set-off system to enable simple settlement of debts to those who are thus mutually obliged and to ensure that both parties acquire fair satisfaction of each debt.

Therefore, for example, in a tripartite relationship in which A has an α claim against B and B has a β claim against C, the set-off between the α claim and the β claim is not allowed. In such a situation, in a case in which C insisted on a set-off between the α claim and the β claim under the agreement with B, the Supreme Court held that such set-off of the two claims did not fall under the set-off stipulated in Art. 92 (1) of the Japanese Civil Rehabilitation Act ^2^.

1-1-2 *Exceptions for assignment of claims*

However, set-off may be permitted if the tripartite relationship arises because of the assignment of claims. In particular, suppose that C originally had an α claim against B and B had a β claim against C, and both parties were mutually indebted. Subsequently, C transferred the α claim to A, resulting in the tripartite relationship.

In this situation, in order for A to exercise the α claim against B, it is basically necessary that either C gives notice of the assignment of the claim to B or that B consents it (JCC Art. 467 (1)). These are the requirements for perfection of the assignment of claim. In addition, even if any of the requirements for perfection is satisfied, B may duly assert against A any event that has taken place with regard to C by the time of completion of the perfection (JCC Art. 468 (1)). On the basis of this discipline, at the time of the assignment of the α claim, the obligor B may exercise the right to set off the α claim of the assignee A and the β claim against the assignor C and extinguish the α claim in the following cases:

Basically, if B acquires β claims before the requirements for perfection of the assignment of α claim between A and C are satisfied, B may exercise the right to set-off against A (JCC Art. 469 (1)). It is necessary for this set-off that neither the eligibility for the set-off had already arisen by the time of completion of the perfection nor that the due date of the β claim was earlier than that of the α claim ^3^.

According to this rule, B cannot exercise the right to set-off against A if B acquires the β claim against C after the requirements for perfection of the assignment have been fulfilled. But the set-off of B is permitted in the following two cases.

The first case is when the β claim has arisen from a cause that existed before the time of the completion of the perfection (JCC Art. 469 (2) (i)). For example, B becomes a guarantor for C’s debt to D by C’s request. Subsequently, C assigns the α claim against B to A, and after the requirement for perfection of the assignment is satisfied, B performs the guarantee obligation to D and acquires a right to reimbursement β against C. In this situation, B can exercise the right to set-off against A because the β claim has arisen from the guarantee contract between B and D by C’s request that existed before the time of the completion of the perfection ^4^.

The second case is one where the β claim has arisen from a contract under which the α claim acquired by the assignee A has arisen (JCC Art. 469 (2) (ii)). For example, a sales contract is concluded between buyer B and seller C after C has assigned the price claim α arising from the future sales contract between B and C to A in advance, and the requirement for perfection of the assignment has been satisfied. But B acquires a claim for damages β on the grounds of non-conformity with the sales contract for the object of sale. The set-off between the α claim and the β claim is available in this case ^5^. The purpose of this discipline is to assure the debtor B of set-off by their future claims. This assurance is expected to encourage B to continue the existing transaction with his creditor C even after the assignment of future claims, and to create an environment in which the continuation of the transaction will increase the likelihood of obtaining future claims and contribute to the interests of assignee A and thereby facilitate C’s financing based on the assignment of future claims ^6^.

However, the set-off is not permitted in any case when B acquires β claim from another person (the proviso of JCC Art. 469 (2)). For example, in the former situation, if it is E, not B, who is requested by C to become a guarantor for C’s debt to D, and after the requirement for perfection of the assignment of α claims from C to A are satisfied, E performs the guarantee obligation to D and acquires a right to reimbursement β against C, then B acquires the β claim from E, in this case, B cannot invoke the set-off between the α claim and the β claim in relation to A.

### 1-2 Existence of obligations

1-2-1 *Principles*

Next, in order to carry out a set-off, which is a reason for extinguishing debt, it is necessary that the eligibility for set-off exists at the time of the manifestation of one party’s intention to set off. In other words, both claims to be set off must be in effect at the time of the exercise of the right to set-off ^7^.

When the active claim is subject to a condition precedent and the condition has not been fulfilled, this claim cannot be set off because it has not yet been effective (JCC Art. 127 (1)) ^8^. In addition, debts that have already been extinguished cannot be extinguished again by set-off. If one of the debts is extinguished owing to reasons such as performance, substitute performance, novation or set-off in a situation where an intention to set off has not been manifested even though an eligibility for set-off has arisen, the set-off becomes impossible thereafter ^9^. It is also not permissible to set off a non-enforceable claim as an active claim. This is because it would have the same result as compulsory performance ^10^.

Furthermore, if the claim existed at the time of the manifestation of intention to set off, but the contract that gave rise to this claim is subsequently subject to voidance or cancellation, it is understood that the claim is retroactively extinguished; that is, it would have never existed at the time of the exercise of the right to set-off, and as a result, the set-off becomes invalid ^11^.

1-2-2 *Set-off by active claim extinguished by prescription*

However, JCC Art. 508 provides an exception to this “existing debt” requirement. According to this article, a claim that has been extinguished by prescription may be set off as an active claim. For example, even if A’s α claim against B is extinguished by prescription after the eligibility for set-off has occurred between the α claim B’s β claim against A, A can defend himself with the set-off against B’s exercise of the β claim. This provision intends to protect the reliance of the parties on the liquidation by set-off, although they may not pay attention to the prescription management as if the claims are substantially extinguished after the set-off becomes eligible ^12^.

In this regard, the reliance on liquidation by set-off is exceptionally protected only when the eligibility for set-off has already occurred between the parties before the period of extinctive prescription of the active claim expired. Therefore, when a party acquires another person’s claim for which the prescription period has already expired and will set it off as an active claim, JCC Art. 508 cannot be applied because the debts lacked reciprocity at the time of the expiration of the prescription period and there was no eligibility for set-off ^13^.

In addition, as will be seen later, the eligibility for set-off requires the arrival of the due dates of both debts. If the due date of the passive claim has not arrived at the time when the period of extinctive prescription of the active claim expired, there is no eligibility for set-off at this point. Even if the creditor of the active claim who asserts the set-off could previously waive the benefit of time stipulation to the passive claim and create an eligibility for set-off, the set-off is not permitted when this waiver had not been implemented by the time of the prescription period, because the eligibility for set-off had not yet been established and there had been no justifiable reliance on liquidation by set-off ^14^.

### 1-3 Homogeneity of obligations

Subsequently, as is clear from the text of JCC Art. 505 (1), the obligations to be set off must have “the same kind of purpose”. Liquidations of mutual debts for different purposes can be achieved by accord and satisfaction (JCC Art. 482), or mutual release (JCC Art. 519).

With regard to the set-off of monetary debts for currencies of different countries, there may be technically doubts about homogeneity. However, if it is possible to convert the amount of one of the debts at the rate in the foreign exchange market and calculate the balance of the debts, the set-off is regarded as available ^15^.

The homogeneity requirement is satisfied if the subject matters of both debts are of the same kind. Since the place of performance is not normally included in the concept of the purpose of obligation, the set-off of debts for which the places of performance are different is not contrary to the homogeneity requirement. Therefore, the first sentence of JCC Art. 507, that states the availability of such a set-off, is only a cautionary provision ^16^. However, if damage is caused by this set-off, the party who made the set-off is obligated to compensate the other party (the second sentence of JCC Art. 507).

### 1-4 Arrival of the due dates of both debts

It is also necessary to set off that the due dates of both debts have already arrived (the main clause of JCC Art. 505 (1)). Therefore, basically, when A sets off his claim against B and B’s β claim against A, both claims must mature at the time. If the set-off by A were available before the α claim would fall due, B, the debtor of this claim, would be unilaterally stripped of the benefit of time stipulation (JCC Art. 136 (1)); otherwise he could refuse to perform the obligation until the due date. In order to protect the benefit of time of B, the arrival of the due dates of both debts is required ^17^.

However, the debtor may waive the benefit of time at any time (JCC Art. 136 (2)). Therefore, when only A’s α claim matures, A, the debtor of the β claim, can immediately set off both claims by waiving the benefit of time for the β claim.

If no time limit is assigned to the performance of an obligation, the obligee can request the performance and the obligor can perform it at any time. In view of this, if no time limit is assigned to A’s α claim, A may set off both claims after the due date of the β claim or after waiving the benefit of time for the β claim. In that case, A doesn’t have to request preliminarily the performance in order to hold B liable for delay with respect to the α claims under JCC Art. 412 (3) ^18^. In addition, if the due date of the α claim has already arrived and no time limit is assigned to the β claim, the set-off is available for A at any time ^19^.

## 2. Negative requirements

### 2-1 Nature of obligations

2-1-1 *Permissivity of set-off*

When all of the positive requirements of the eligibility for set-off described above are satisfied, the set-off is regularly available for parties. However, if there are other grounds for restricting the set-off, it is prevented.

First, if the nature of the obligation does not allow for set-off, it cannot be set off (the proviso of JCC Art. 505 (1)). The nature of the obligation that makes set-off possible is sometimes referred to as “permissivity of set-off” ^20^.

Obligations that lack the permissivity of set-off typically are obligations of omission, such as not making noise, not setting up vibrations, not stinking, etc., or non-competition, or obligations to provide services when the obligee needs the action of others, such as third-party assessment or tasks that are difficult for one person to do ^21^. In these cases, actual performance is necessary and a set-off is regarded as impermissible because it would prevent the realization of the purpose of the obligation.

2-1-2 *Set-off by active claims to which the right of defense is attached*

In relation to the permissivity of set-off, a right of defense attached to the active claim is also considered as a reason for limitation of set-off. The reason is that the set-off by such an active claim would deprive unilaterally the other party of the right of defense ^22^. The rights concerned are, for instance, the guarantor’s right of defense of demand and debtor’s financial resources (JCC Art. 452 and Art. 453) ^23^, the principal obligor’s right to demand the guarantor requested by him/her to provide security against the guarantor’s right to reimbursement (JCC Art. 461 (1)) ^24^ and the right of defense of simultaneous performance (JCC Article 533) ^25^. Meanwhile, a person who has the right of defense may freely waive it. Therefore, even if a right of defense is attached to the passive claim, there is no hindrance for the debtor to waive the right of defense and set off the claim ^26^.

However, there is an important exception to this set-off limitation. That is the case in a contract for work to set off a claim of a party ordering work for damages in lieu of the repair of defects and a contractor’s claim for remuneration. The right of defense of simultaneous performance is attached to both claims in this case. However, the Japanese Supreme Court has judged that the set-off of both claims is permitted. From its perspective, there is no special benefit that requires actual performance of these on the grounds that there is a consideration between the contractor’s obligation to deliver the object, which gives rise to the right to claim damages, and the obligation of the other party to pay the remuneration and that the right to claim damages in lieu of the repair of defects has the function of substantially and economically reducing the remuneration and providing an equivalent relationship between the mutual obligations of the parties. Then there is no disadvantage due to the loss of the right of defense as a consequence of the set-off. Instead, the liquidation by the set-off is convenient and fair for both parties and simplifies the legal relationship ^27^.

### 2-2 Manifestation of intention to limit a set-off

Second, it is permissible for parties to prohibit or restrict a set-off (JCC Art. 505(2)). Even if a set-off is not prohibited by law, it can be limited in this way when the parties require actual performance.

An intention to limit a set-off may be manifested by an agreement of the parties when the cause of the obligation is a contract, or at the time of the act when it is a unilateral juridical act ^28^. The parties can enter the agreement to limit a set-off after the obligation has arisen ^29^.

However, the limitation of set-off may be duly asserted against a third party only if the third party knew it or did not know it owing to gross negligence (JCC Art. 505 (2)). If a prohibition of set-off is agreed upon for A’s α claim against B, then in order for B or A to assert non-set-off against C, who has acquired the α claim from A, or D, who has assumed the obligation with respect to the α claim in order to release B, C and D must be aware of this agreement or grossly negligent in not recognizing it. Nonetheless, if A wants to preserve the benefit of obtaining actual performance by the assumption of obligation releasing B, it is necessary not to agree or consent to D’s assumption (JCC Art. 472 (2) and (3)). ^30^

### 2-3 Claims for damage due to tort, death and injury

Third, set-off is prohibited when certain claims for damage are laid as a passive claim. This prohibition applies to the right to claim damages based on a tort committed in bad faith (JCC Art. 509 (i)) and the right to claim damages for death or injury to person (JCC Art. 509 (ii)). When A has a claim against B and B has any of these rights, A cannot set off B’s right.

The set-off prohibition for “tort committed in bad faith” applies to situations where B’s creditor A commits a tort against B. The main points of the prohibition in such a situation are not only that the victim B must be compensated by actual performance, but also that the perpetrator A deserves no benefit by set-off, and that it is necessary to prevent the creditor A from tortious acts against the debtor B ^31^. With respect to the prevention of the inducement to tortious acts, retaliatory acts by A, who cannot accept performance from B, are assumed. The “bad faith” in the provision is explained as “the intention to aggressively harm others” ^32^, and mere normal intention is not enough ^33^. Furthermore, the liability of structure on land (JCC Art. 717) is not subject to the prohibition of set-off because it is not based on the subjective intention of a person and “bad faith” is not expected ^34^. Also, there is controversy as to whether the prohibition of set-off may be applied not only in the case of tort but also in the event of default of obligation. According to the wording of the provision, the prohibition of set-off does not extend in the case of default on both sides ^35^. But there is an opposing view that it should be prohibited to set off a claim for damages based on “default with the intention to aggressively harm the creditor” ^36^.

The reason why set-off is prohibited in the case of “death or injury to person” is primarily because there is a high need for relief of the victim by actual performance ^37^, and secondarily because the perpetrator deserves no benefit by set-off ^38^. So, the severity of the violated interests of the victim is of crucial significance to this prohibition. Therefore, no regard is given to the circumstances related to the perpetrator’s act, such as whether the victim’s right to claim damages is caused by tort or default, whether the tort is due to negligence, “bad faith”, or whether it is a matter of liability of structure on land ^39^.

Thus, both of these prohibitions of set-off are primarily aimed at providing actual performance to the victim, i.e., protecting the victim themself. It is not excluded that the victim waives such protection and sets off the claim for damages as an active claim ^40^. According to this purpose, if A’s act of misconduct is against C and not B, and B acquires the right to claim damages from C, B themself is not the victim and does not need to be protected by the prohibition of set-off. Therefore, it is not precluded that A set off B’s claim for damages as a passive claim (the proviso of JCC Art. 509). However, the lifting of this set-off ban is only applicable to cases where B acquires the passive claim. When the claim is transferred from C to B in the form of a comprehensive succession such as an inheritance or a corporate merger, the set-off prohibition still applies ^41^.

### 2-4 Claims exempt from attachment

Fourth, set-off is not permitted if the passive claim is a claim exempt from attachment (JCC Art. 510) ^42^. The prohibition of attachment of claims is provided when actual performance is indispensable for the creditor. The reason for this prohibition is that the purpose of prohibiting the attachment of claims comes to nothing due to set-off ^43^. Thus, since the provision is intended to protect the creditor of a claim exempt from attachment, it is not prohibited for this creditor to waive the protection and to set off the claim as an active claim.

### 2-5 Set-off of the active claim acquired after the attachment of the passive claim

2-5-1 *Principles*

Fifth, when a claim has been attached and the debtor of it acquires another claim against the creditor after the attachment, the debtor may not assert the set-off of both claims as defense against the attaching obligee (JCC Art. 511 (1)). In other words, for the debtor A, against whom the creditor B has the β claim, the set-off of the β claim as a passive claim is in principle not available when A acquires the α claim as an active claim against B after the β claim was seized by C.

Conversely, when A’s acquisition of the α claim precedes C’s attachment of the β claim, A may duly assert the set-off of both claims as defense against C (JCC Art. 511 (1)). Besides the requirement of an active claim acquired before the attachment of passive claim, no other factors about the claims such as temporal order of due dates, transactional connection or types of transaction are taken into consideration ^44^. However, eligibility for set-off is still necessary. If the β claim matures before the α claim, A may not assert the set-off against C until the α claim matures, and C can exercise the β claim against A.

2-5-2 *Set-off of the active claim based on the cause before the attachment*

There is an exception to this prohibition. Even if A acquires the α claim after the attachment of the β claim by C, A may duly assert a claim set-off against C when the cause that gives rise to the α claim existed before C’s attachment (the main clause of JCC Art. 511 (2)). For example, B has a β claim against A. And A becomes a guarantor of B’s debt to D by request from B. Subsequently, C attaches the β claim, and then A fulfills the guarantee obligation to D and acquires the α claim for indemnification against B. In this case, A acquires the α claim indeed after the C’s attachment of the β claim. But since the guarantee contract between D and A by request from B, which is the cause of the α claim, was concluded before the attachment, A may assert the set-off against C ^45^.

However, even if A’s α claim arises owing to a cause that existed before the attachment of the β claim by C, A cannot assert the set-off against C when A acquired the α claim from another person after the attachment (proviso of JCC Art. 511 (2)). For example, B has a β claim against A, and E becomes a guarantor of B’s debt to D by request from B. Subsequently, C attaches the β claim, and then E assigns to A the α claim for indemnification against B that E will acquire if E fulfills the guarantee obligation in the future. Furthermore, E fulfills the guarantee obligation to D and A acquires the α claim. In this case, although the guarantee contract between D and E, which is the cause of the α claim, was concluded before the attachment, A cannot assert the set-off against C because it was after the attachment that A acquired the α claim from E ^46^.

### 2-6 Prohibition of set-off under the law

Sixth, there are legal provisions that prohibit set-off.

JCC Art. 677 is interpreted as a provision that prohibits a debtor of a partnership from set-off who is personally a creditor of one of the partners. This is because the partnership property would be reduced in order to fulfill the individual debt of the partner, if the debtor of the partner could set off the claim of the partnership against them and their claim against the partner ^47^. In addition, a partner may not independently exercise their interest in the right with regard to a claim that is included in the partnership property (JCC Art. 676 (2)). Therefore, a partner, against whom a debtor of a partnership, cannot set off the claim of the partnership against the debtor and the claim of the debtor against them ^48^.

There are such provisions in laws other than the JCC: the prohibition on the grounds that the trust property is independent of the personal property of the trustee (Art. 22 of the Trust Act) ^49^, the prohibition in order to require investors to contribute actual assets under the principle of adequacy of equity capital (Art. 208 (3) and Art. 281 (3) of the Companies Act) ^50^, and the prohibition with the aim of preventing de facto forced labor and the inducement of body restraint based on previous loans (Art. 17 of the Labor Standards Act and Art. 35 of the Mariners Act) ^51^.

## 3. Requirements for set-off in bankruptcy proceedings

### 3-1 Overview

When a court commences bankruptcy proceedings, with the aim of equality of treatment for creditors, the bankruptcy creditors are prohibited from exercising their individual rights in principle (JBA Art. 100 (1)). On the other hand, the set-off has a similar function to a security interest when a debtor does not fulfill their obligation. The need for the collection of claims with security interest is greatest particularly when the debtor goes bankrupt. Therefore, in bankruptcy proceedings, the exercise of the right to set-off is widely permitted with the aim of protecting the creditor’s expectation of the security function of set-off. This paper mainly discusses the rules regarding the set-off by bankruptcy creditors in bankruptcy proceedings as liquidation-type proceedings.

In view of the security function of set-off, bankruptcy creditors are basically allowed to exercise their right to set-off without bankruptcy proceedings as the exception to the prohibition of exercising individual rights (JBA Art. 67 (1)). However, from the standpoint of other bankruptcy creditors, such set-offs allow for preferential debt collection by the bankruptcy creditor exercising their right to set-off, even though the passive claims of the bankrupt belong to the bankruptcy estates, which should be distributed equally among the creditors. Therefore, set-off is prohibited in certain cases where it is regarded as unfair from the viewpoint of equality of treatment for bankruptcy creditors (JBA Art. 71 (1) and Art. 72 (1)). In addition, even if a set-off falls formally under any one of the prohibitions, the prohibition can be lifted in certain situations (JBA Art. 71 (2) and Art 72 (2)) ^52^. The following is a look at the rules regarding set-off under bankruptcy law that form this hierarchical structure.

### 3-2 Relaxation of requirements

Bankruptcy creditors are allowed to exercise their right to set-off without bankruptcy proceedings by their bankruptcy claims as an active claim and claims of the bankrupt against themselves as a passive claim that there were at the commencement of bankruptcy proceedings. Even in this case, it is basically required that the suitability for set-off stipulated in the Civil Code already exists. However, as a result of the rules of bankruptcy law, the requirements for set-off under the Bankruptcy Act applicable in crisis time may be more relaxed than those under the Civil Code applicable in normal times.

3-2-1 *Relaxation of active claims*

One of the requirements for eligibility for set-off is the homogeneity of debts. As a general rule, when the passive claim is a monetary claim, the active claim must be also a monetary claim. However, in bankruptcy proceedings, even if the bankruptcy claim is a non-monetary claim, the amount of the claim is calculated based on the valuation at the time of commencement of the proceedings, and the claim is regarded as a monetary claim (monetization, JBA Art. 103 (2) (i)). From the viewpoint of eligibility for set-off, the homogeneity of debts is arranged as a result of the monetization of non-monetary claims. This rule allows bankruptcy creditors with non-monetary claims to set off monetary claims against themselves as passive claims in bankruptcy proceedings. However, the non-monetary claim subject to this monetization must be a property claim that can be evaluated financially. For example, it applies to the rights to demand delivery of goods, provision of services and fungible acts. On the other hand, the rights to demand non-fungible acts or omission are not property claims because they are unsuited for financial evaluation. Thus, they do not fall under bankruptcy claims and are not subject to monetization ^53^.

And under ordinary circumstances, the eligibility for set-off requires that both claims mature. If the due date of the active claim has not arrived, the creditor cannot set it off. However, when bankruptcy proceedings are commenced, the bankruptcy claims with due dates are deemed to have become due at the time of commencement of bankruptcy proceedings, even if they have not yet matured (JBA Art. 103 (3)). Consequently, even when the bankruptcy claims have not yet matured before the bankruptcy proceedings are commenced, the creditors can set them off (the first sentence of JBA Art. 67 (2)). ^54^

In addition, the existence of claims is necessary for the eligibility for set-off. If the bankruptcy creditor holds a claim subject to a condition precedent or a claim which may arise in the future and the claim has not yet arisen, there is no eligibility for set-off. But the Bankruptcy Law assumes that the claim and the eligibility for set-off can arise between the commencement of bankruptcy proceedings and the period of exclusion concerning a final distribution and will assure the bankruptcy creditor of set-off in this situation. In concrete terms, when the bankruptcy creditor pays their debt to the bankrupt, they may request a contractual deposit of the amount of payment up to the amount of their claim in order to set off their debt later (the first sentence of JBA Art. 70). If their bankruptcy claim arises after the deposit, the deposited amount will be returned to the bankruptcy creditor, and they may exercise the right to set-off to extinguish their debt ^55^.

Meanwhile, when the bankruptcy creditor holds a claim subject to condition subsequent, there is no hindrance to setting it off as an active claim from the viewpoint of eligibility for set-off because the claim exists until the condition is fulfilled (the first sentence of JBA Art. 67 (2)). However, if the conditions are fulfilled before the period of exclusion concerning a final distribution, the eligibility for set-off will disappear retroactively and the set-off deserves very little protection. Therefore, in order to ensure the performance of the obligation related to the passive claims belonging to the bankruptcy estate at the time of the fulfillment of the condition in the future, when the bankruptcy creditor sets off their claim subject to condition subsequent as an active claim, they must provide security for or make a contractual deposit of the amount of their debt to be extinguished by the set-off (JBA Art. 69) ^56^.

3-2-2 *Relaxation of passive claims*

When a bankruptcy creditor will set off a claim belonging to the bankruptcy estate as a passive claim in bankruptcy proceedings, the set-off is available even if the claim belonging to the bankruptcy estate is subject to a due date or condition (the second sentence of JBA Art. 67 (2)). If the due date of the passive claim has not yet arrived, the bankruptcy creditor may create an eligibility for set-off by waiving the benefit of the time stipulation attached to their debt (JCC Art. 136 (2)). Therefore, there is no special meaning in the treatment for a claim subject to a due date. On the other hand, with regard to a claim subject to condition, there is a dispute about whether the provision of the second sentence of JBA Art. 67 (2) is an exception under the Bankruptcy Law to the general principle of the Civil Code ^57^ or it is available too on the Civil Code to waive the benefit of non-fulfillment of the condition ^58^ ^59^.

Meanwhile, unlike with bankruptcy claims, the provision on the monetization of non-monetary claims (JBA Art. 103 (2) (i)) does not apply to claims belonging to the bankruptcy estate against bankruptcy creditors. Therefore, if a bankruptcy creditor who holds a monetary claim as bankruptcy claim will set off a claim belonging to the bankruptcy estate against them, this claim must be of the same type as their active claim, that is, a monetary claim ^60^.

### 3-3 Prohibition of set-off

In this way, the right to set-off of bankruptcy creditors is widely protected under the Bankruptcy Law. But such a set-off also has a similar function to a security interest that allows for preferential debt collection by the bankruptcy creditor exercising their right to set-off. Therefore, in certain cases where the protection of the benefit by set-off shows unfair favoritism toward the bankruptcy creditor exercising the right to set-off, their set-off is prohibited in order to ensure equality of treatment among the bankruptcy creditors ^61^.

In this section, we will show the prohibition rules in the situation where A who holds an α claim as a bankruptcy claim against the bankrupt B will set off a β claim belonging to the bankruptcy estimate against A.

3-3-1 *Rules on passive claims*

3-3-1-1 Incurring of debts after the commencement of bankruptcy proceedings

To begin with, the Bankruptcy Law prohibits set-off with the object of how the bankruptcy creditor has incurred debt.

First, when the bankruptcy creditor A incurs a debt to the bankruptcy estate after the commencement of bankruptcy proceedings against B, A cannot set it off (JBA Art. 71 (1) (i)).

After this base point in time, individual payments to the bankruptcy creditors are impermissible in principle (JBA Art. 100 (1)). If the set-off based on changing situations after this time point were permitted, it would provide a back door for bankruptcy creditors to receive individual payments. If the set-off based on the par value of the bankruptcy claim were available, although this claim is certain to have a less economic value than the par value due to the order to commence bankruptcy proceedings, it would be substantially the same as creating a security interest for the specific bankruptcy claim after the commencement of bankruptcy proceedings and would prejudice equality among the bankruptcy creditors. These are the reasons for the prohibition of set-off ^62^.

For example, if the bankruptcy creditor incurs a debt due to a juridical act with the bankruptcy trustee after the commencement of bankruptcy proceedings ^63^ or the exercise of a right of avoidance by the bankruptcy trustee ^64^, the set-off of this debt is not available.

However, when a claim belonging to the bankruptcy estate is subject to a condition precedent and the condition is fulfilled after the commencement of bankruptcy proceedings, the bankruptcy creditor who is the debtor of the claim is not prohibited from setting it off as a passive claim because a bankruptcy creditor may set off a claim belonging to the bankruptcy estate subject to condition (the second sentence of JBA Art. 67 (2)) ^65^.

3-3-1-2 Incurring of debts based on the contract after becoming unable to pay debts

Second, A enters into a contract before the commencement of bankruptcy proceedings against B, but after B became unable to pay debts. And A knows B’s inability of payment at the time of conclusion of the contract. If this contract is concluded with B and aimed at disposing of B’s property with the intent to offset any debt to be incurred by A under the contract exclusively against A’s bankruptcy claims, A is prohibited from setting off the debts after the commencement of bankruptcy proceedings against B. The prohibition applies too when the debt which A incurs under the contract was originally owed by another person to B (JBA Art. 71 (1) (ii)).

The time that the future bankrupt B becomes unable to pay debts is set as the base in the provision because B cannot pay even debts that are due and equality of treatment among B’s creditors should be required to the same extent as after B’s suspension of payment or the commencement of bankruptcy proceedings against B. If the set-off by A were permitted on these situations, it would be similar to giving A a security interest contrary to the principle of creditors’ equality ^66^.

With regard to the relationship between A and B after B becomes unable to pay debts, it can be regarded as unfair favoritism similar to accord and satisfaction that A acquires B’s property and can set off the debt which is the consideration of it. On the other hand, it is also necessary to give care to the continuity of B’s business through ordinary transactions with A. Therefore, the applicability of “a contract with the bankrupt for disposing of the bankrupt’s property with the intent to offset any debt to be incurred by the bankruptcy creditor under the contract exclusively against bankruptcy claims” should be determined from the viewpoint of whether or not the contract between A and B evades the avoidance of provision of security to specific creditors (JBA Art. 162 (1) (i)), taking into account various circumstances before and after its conclusion ^67^.

In addition, with regard to a contract to incur an existing debt owed by another person to B, the assumption of the debt after recognizing B’s inability to payment is considered to be for the purpose of debt collection and the set-off of the assumed debt is prejudicial to the equality among creditors. Even if the set-off is prohibited because of A’s assumption of the debt, ordinary transactions between A and B could not shrink. Therefore, the set-off by A is prohibited regardless of the cause or purpose of the assumption of the debt ^68^.

3-3-1-3 Incurring of debts after suspension of payment

Third, when A incurs a debt to B after B suspended payments and A was aware of B’s suspension of payment at this time, A cannot set it off after the commencement of bankruptcy proceedings against B (the main clause of JBA Art. 71 (1) (iii)).

Because A is aware of B’s suspension of payment and also recognizes a high probability of B’s insolvency, A’s acquisition of the right to set-off is regarded as detrimental to equality among the bankruptcy creditors ^69^.

However, even if A is aware of B’s suspension of payment at the time of incurring the debt, A is exempt from the prohibition of the set-off when they prove that B was not objectively insolvent at the time (proviso of JBA Art. 71 (1) (iii)) ^70^.

3-3-1-4 Incurring of debts after a petition to commence bankruptcy proceedings

Fourth, when A incurs a debt to B after a petition to commence bankruptcy proceedings against B is filed and A is aware of the petition at that time, the set-off of the debt is not available for A (JBA Art. 71 (1) (iv)).

After the filing of a petition for the commencement of bankruptcy proceedings, creditors who are aware of this should be restricted from individual debt collection. In this prohibition of set-off, it does not matter whether B was objectively insolvent or not ^71^.

3-3-2 *Rules on active claims*

3-3-2-1 Acquisition of claims after the commencement of bankruptcy proceedings

On the other hand, the Bankruptcy Law prohibits set-off with the object of how the bankruptcy creditor has acquired claim.

First, when A acquires a bankruptcy claim from another person after the commencement of bankruptcy proceedings against B, A cannot use the claim for set-off (JBA Art. 72 (1) (i)).

When an eligibility for set-off is created after the commencement of bankruptcy proceedings, the set-off based on it is impermissible for the purpose of bankruptcy proceedings because of the principle of fairness and equality among bankruptcy creditors ^72^.

Meanwhile, there is a case in which the judicial precedent applies this provision by analogy: In particular, B has a β claim against A, and A becomes a guarantor of B’s debt to C under a contract between A and C without a request from B. Subsequently, the bankruptcy proceedings are commenced against B, and then A fulfills the guarantee obligation to C and acquires the α claim for indemnification against B. In this case, the α claim of A is a bankruptcy claim. But A is a guarantor without entrustment and acquired the α claim after the commencement of bankruptcy proceedings. If A were nevertheless permitted to set off the α claim and the β claim, it would be equal to permitting the creation of a claim which may be treated preferentially during bankruptcy proceedings, independently of B’s intention or any statutory cause. A’s expectation for such a set-off is not reasonable. In addition, such a set-off is similar to a set-off which a debtor to the bankrupt seeks by acquiring another person’s claim after the commencement of bankruptcy proceedings. Both set-offs are impermissible for the purpose of bankruptcy proceedings because of the principle of fairness and equality among bankruptcy creditors. Therefore, “where a guarantor without entrustment pays a debt of the principle debtor after the commencement of bankruptcy proceedings against the principal debtor in accordance with the contract of guarantee concluded before the commencement of the proceedings, a set-off sought by the guarantor, on the basis of his/her right to indemnification acquired through such payment, against the claim held by the bankrupt (the principal debtor) against the guarantor, is impermissible pursuant to Article 72, paragraph (1), item (i) of the Bankruptcy Act as applied by analogy”. However, on the other hand, if A were a guarantor entrusted by the principal debtor B, the set-off of the α claim which A acquired by the payment to C after the commencement of bankruptcy proceedings would be “reasonable and deserves to be protected under Article 67 of the Bankruptcy Act” ^73^.

3-3-2-2 Acquisition of claims after becoming unable to pay debts

Second, when A acquires a claim against B after B became unable to pay debts and A was aware of B’s inability of payment at the time of acquisition, A cannot set off the claim after the commencement of bankruptcy proceedings against B (JBA Art. 72 (1) (ii)).

In this case, the principle of equal treatment among bankruptcy creditors takes precedence over A’s expectation for a set-off. Unlike the similar prohibition of set-off by debts incurred after becoming unable to pay debts (JBA Art. 71 (1) (ii)), there is no limitation according to A’s purpose of acquiring the claims or the content of the contract that gave rise to the claim, because A undertakes the risk of no payment from B despite B’s inability by themself and there is no need to give care to ordinary transactions between A and B ^74^.

3-3-2-3 Acquisition of claims after suspension of payment

Third, when A acquires a claim against B after B suspended payments and A was aware of B’s suspension of payment at the time of acquisition, the set-off of the claim is not available for A (the main clause of JBA Art. 72 (1) (iii)).

As with the rules on debts incurred after suspension of payment (JBA Art 71 (1) (iii)), this prohibition of set-off does not apply if A proves that B was not objectively insolvent at the time of acquisition of the claim (proviso of JBA Art. 72 (1) (iii)).

3-3-2-4 Acquisition of claims after a petition to commence bankruptcy proceedings

Fourth, when A acquires a claim against B after a petition to commence bankruptcy proceedings against B is filed and A is aware of the petition at the time of acquisition, the set-off of the claim by A is prohibited (JBA Art. 72 (1) (iv)).

It is a provision to the same effect as the prohibition of set-off in the case of debt incurred in the same situation (JBA Art. 71 (1) (iv)).

### 3-4 Lifting the prohibitions of set-off

3-4-1 *Incurring of debts and acquisition of claims due to statutory causes*

The Bankruptcy Law further lifts the prohibitions of set-off, when the debt incurred or acquisition of claims formally subject to one of the prohibitions is based on certain causes. This is because the expectation for set-off deserves to be protected in the relevant situation.

First, when the suspect incurring of debts or acquisition of claims are based on statutory causes, the set-off of these is available (JBA Art. 71 (2) (i) and Art. 72 (2) (i)).

In this case, the prohibitions of set-off are lifted because the eligibility for set-off here is not intentionally created by the bankruptcy creditor, the bankrupt or a third party. Therefore, even if a bankruptcy creditor incurs a dept or acquires a claim due to statutory causes, their set-off should not be permitted when their intention may intervene as in the case of company split or merger ^75^.

3-4-2 *Incurring of debts and acquisition of claims due to causes prior to recognition of the determinate facts*

Second, when the suspect incurring of debts or acquisition of claims are based on a cause that has occurred before the bankruptcy creditor comes to know the bankrupt’s inability of payment, their suspension of payment or the petition to commence bankruptcy proceedings against them, the set-off of these is not prohibited (JBA Art. 71 (2) (ii) and Art. 72 (2) (ii)).

When either an active claim of the creditor or a passive claim of the bankrupt had already occurred and there was already a cause to create the other before the crisis time, the bankruptcy creditor’s expectation for set-off is deemed reasonable and secured as is the case in a security interest ^76^. According to such a concept, it is often said that the cause here must justify directly and concretely the expectation for set-off of the incurred debt or the acquired credit ^77^.

For example, financial institution A has entered into a transaction agreement with counterparty B before A awakes to B’s insolvency. The agreement includes clauses that A may collect or retire B’s bills in A’s possession in the event of B’s default and provide the collected amount for B’s debt. Under the clauses, A is entrusted by B to collect bills and obtains endorsements. Then, after A knows of B’s insolvency, but before the commencement of bankruptcy proceedings against B, A collects the bills and falls into debt to deliver the collected amount to B. In such a situation, A’s expectation for set-off of the debt incurred after the awareness of B’s insolvency deserves protection and the transaction agreement is said to be a cause here ^78^.

In addition, when A acquires the right to claim payment of bills against B under the previous bill discounting agreement with B after A was aware of B’s suspension of payment, or when A and B are joint and several obligors and A acquires the right to reimbursement against B after A knew of the petition to commence bankruptcy proceedings against B, the bill discounting contract or the joint and several obligation can be considered as the cause ^79^.

However, even if financial institution A has concluded a current account agreement or a savings account agreement with B before the crisis period of B, the agreements do not fall under “cause”. This is because it is uncertain whether A incurs a debt under the agreement. Therefore, A’s expectation for set-off of the debt lacks concreteness ^80^.

Furthermore, the prohibitions of set-off are not lifted in the following case too: B enters into an agreement with a bank A to entrust A to conduct management of beneficial interests in an investment trust. According to the agreement, if B executes the cancellation of the trust agreement concerning the beneficial interests, the trust company C should transfer the cancellation money to A and A must pay the money to B. After B suspends payments, A files a request for execution of cancellation with respect to the beneficial interests with C, based on the obligee’s subrogation right (JCC Art. 423) and on behalf of B, in order to preserve their claim against B. Then, A receives the cancellation money from C and assumes the obligation to pay the money to B. In this situation, it was uncertain whether A incurred the debt against B or not, because B could transfer the beneficial interests to other book-entry accounts even during the time when A managed the beneficial interests under the agreement. Therefore, A’s expectation for set-off of the debt under the agreement was not reasonable ^81^.

In this way, even if the legal relationship that gives rise to the passive claims or active claims has formally arisen before the bankruptcy creditor becomes aware of bankrupt’s inability of payment, etc., the prohibition of set-off may not be lifted when there are circumstances in which the expectation for set-off is not reasonable. However, in order for such an expectation to be evaluated as reasonable, it is not necessary that the active claim and the passive claim are based on the same contract ^82^.

3-4-3 *Incurring of debts and acquisition of claims due to causes not less than one year prior to the filing of the petition for commencement of bankruptcy proceedings*

Third, when the suspect incurring of debts or acquisition of claims are based on a cause that occurred not less than one year before the filing of the petition for commencement of bankruptcy proceedings, the set-off of these is permitted (JBA Art. 71 (2) (iii) and Art. 72 (2) (iii)).

Otherwise, a creditor could not judge indefinitely whether they could set off their debt or claim when they had incurred the debt or acquired the claim after their debtor had become unable to pay debts or suspended payment. It is harmful to the credibility and safety of transactions to place the creditor in such a precarious position for more than one year ^83^.

3-4-4 *Acquisition of claims due to a contract with the bankrupt*

Fourth, when a debtor of a bankrupt acquires a claim on the basis of a contract with the bankrupt, they may set off their debt and claim, even if the contract is concluded in the crisis period of the bankrupt (JBA Art. 72 (2) (iv)).

For example, B has a claim against A. In this situation, A can expect that their future claims against B would be secured by the set-off against B’s cross-claim. So that means that A is in much the same position as if they has excess security interest against B. When A acquires a claim against B due to a contract with B and sets off the claim and B’s cross-claim, it is equal to a use of the security interest. Even if the contract is concluded in B’s crisis period, the set-off by A decreases their bad debt risk to lower than before, because A has not been an unsecured creditor, so to speak ^84^.

According to the provision, the scope of JBA Art. 72 (1) (ii) to (iv) is practically limited to the case where a debtor of a bankrupt acquires a bankruptcy claim of another person ^85^.

But this lifting of the prohibitions of set-off is not applicable when a creditor of a bankrupt incurs a dept on the basis of a contract with the bankrupt. It is essentially similar to an acquisition of new security interest.

## 4. Conclusion

As previously explained, the basic requirements for set-off in Japanese law have many things in common with other civil law countries. But it is observed characteristically that the expectation for set-off is widely protected and the set-off functions just as mighty security interests.

For example, B’s passive claim β against A is assigned to C or seized by C before an eligibility for set-off arises. In German law, A may assert the set-off of their active claim α and the β claim against C, only when A acquired the α claim before the assignment or the attachment and the α claim matures before the β claim (BGB Art. 392 and Art. 406). Comparatively speaking, in Japanese law, it does not matter which of the claims matures first when A acquires the α claim before the assignment or the attachment (JCC Art. 469 (1) and Art. 511 (1)) ^86^. And what is more, A may set off both claims in some situations, even when they acquire the α claim after the assignment or the attachment (JCC Art. 469 (2) and Art. 511 (2)) ^87^.

In addition, on the German Insolvency Act (InsO), a bankruptcy creditor, who has not yet a right to set-off before the commencement of bankruptcy proceedings, cannot set off their claim in the proceedings before an eligibility for set-off arises (InsO Art. 94 and the first sentence of Art. 95 (1)) or when a passive claim of the bankrupt matures before that (the third sentence of InsO Art. 95 (1)). Besides, the prohibitions of set-off can be lifted only in limited circumstances where the German Credit System Act applies (InsO Art. 96). On the other hand, JBA permits set-off immediately, even if active claims of bankruptcy creditors are not monetary or do not mature yet (the first sentence of JBA Art. 67 (2)), and if passive claims belonging to the bankruptcy estate are subject to a due date or condition (the second sentence of JBA Art. 67 (2)) ^88^. Moreover, the prohibitions of set-off are lifted more broadly (JBA Art. 71 (2) and Art. 72 (2)) ^89^.

The characteristics of Japanese law are strengthened by the revision of the JCC in 2017. The strong protection of set-off contributes to the stabilization and development not only of financial transactions but also of all other continuous transactions.

## Ethics and consent

Ethical approval and consent were not required.

## Data availability statement

No data are associated with this article.

## Footnotes

^2^
Judgement of Supreme Court, 8 July 2016, Minshu Vol. 70, No. 6, p. 1611. However, there has long been the opinion that some set-off among the three parties should be allowed under certain conditions.

^3^
Hiroyasu Nakata, *General Theory of Obligation Law* (4th ed., Tokyo: Iwanami Shoten, 2020), p. 657. This discipline is based on the views of judicial precedents before the revision of the JCC in 2017. See Judgement of Supreme Court, 8 December 1975, Minshu Vol. 29, No. 11, p. 1864. See also BGB Art. 406, FCC Art. 1347-7 and Art. 1348-1 (3).

^4^
Nakata (n. 3), p. 657.

^5^
Justice Ministry’s Legislative Council Civil Code (Obligation Law) Subcommittee Document 74A, p. 15; Nakata (n. 3), p. 659.

^6^
Justice Ministry’s Legislative Council Civil Code (Obligation Law) Subcommittee Document 74A, pp. 14 f.; Nakata (n. 3), pp. 659 f.

^7^
Yoshio Nakai, ‘Article 505 Civil Code’ in Tetsu Isomura (ed.), *Commentary of the Civil Code (12) Obligation (3)* (Tokyo: Yuhikaku, 1970), p. 388.

^8^
Yoshio Shiomi, *New General Theory of Obligation Law II* (Tokyo: Shinzansha, 2017), p. 273.

^9^
Judgement of Supreme Court, 10 July 1979, Minshu Vol. 33, No. 5, p. 533; Sakae Wagatsuma, *New Revised General Theory of Obligation Law* (Tokyo: Iwanami Shoten, 1964) pp. 350 f.; Shouzou Inui, ‘Article 506 Civil Code’ in Tetsu Isomura (ed.), *Commentary of the Civil Code (12) Obligation (3)* (Tokyo: Yuhikaku, 1970), pp. 419 f.; Fujio Oho, *General Theory of Obligation Law* (new ed., Tokyo: Yuhikaku, 1972), p. 424; Masamichi Okuda, *General Theory of Obligation Law* (enlarged ed., Tokyo: Yuyusha, 1992), p. 574; Kikuo Ishida, ‘Set-Off’ in Ryohei Hayashi et al., *General Theory of Obligation Law* (3rd ed., Tokyo: Seirin Shoin, 1996), p. 353; Shiomi (n. 8), p. 275; Nakata (n. 3), p. 461 and pp. 468 f.

^10^
Wagatsuma (n. 9), p. 324; Shiomi (n. 8), p. 295; Hiroyuki Hirano, *General Theory of Obligation Law* (2nd ed., Tokyo: Nippon Hyoronsha, 2023), pp. 511 f.

^11^
Judgement of the Great Court of Judicature, 7 April 1920, Minroku Vol. 26, p. 458; Sakae Wagatsuma, *Special Theory of Obligation Law vol. 1* (Tokyo: Iwanami Shoten, 1954), p. 193; Wagatsuma (n. 9), p. 324; Nakai (n. 7), p. 388.

^12^
Wagatsuma (n. 9), p. 325; Nakai (n. 7), p. 389; Shouzou Inui, ‘Article 508 Civil Code’ in Tetsu Isomura (ed.), *Commentary of the Civil Code (12) Obligation (3)* (Tokyo: Yuhikaku, 1970), pp. 423 f.; Shiomi (n. 8), p. 277; Nakata (n. 3), p. 469.

^13^
Judgement of the Great Court of Judicature, 28 September 1940, Minshu Vol. 19, p. 1744; Judgement of Supreme Court, 14 April 1961, Minshu Vol. 15, No. 4, p. 765; Nakai (n. 7), p. 389; Inui (n. 12), p. 425; Okuda (n. 9), p. 574; Ishida (n. 9), p. 336; Shiomi (n. 8), pp. 279 f.; Nakata (n. 3), p. 469.

^14^
Judgement of Supreme Court, 28 February 2013, Minshu Vol. 67, No. 2, p. 343; Shiomi (n. 8), p. 273.

^15^
Shiomi (n. 8), p. 271.

^16^
Shouzou Inui, ‘Article 507 Civil Code’ in Tetsu Isomura (ed.), *Commentary of the Civil Code (12) Obligation (3)* (Tokyo: Yuhikaku, 1970), p. 421.

^17^
Nakata (n. 3), p. 470.

^18^
Judgement of the Great Court of Judicature, 19 November 1942, Minshu Vol. 21, p. 1075; Wagatsuma (n. 9), p. 327; Nakai (n. 7), p. 395; Okuda (n. 9), p. 574.

^19^
Judgement of the Great Court of Judicature, 8 September 1934, Minshu Vol. 12, p. 2124; Wagatsuma (n. 9), p. 328; Nakai (n. 7), p. 396.

^20^
Nakai (n. 7), p. 396.

^21^
Wagatsuma (n. 9), p. 329; Nakai (n. 7), p. 396; Okuda (n. 9), p. 574; Nakata (n. 3), p. 470.

^22^
Judgement of the Great Court of Judicature, 13 November 1914, Minroku Vol. 20, p. 922; Judgement of the Great Court of Judicature, 24 October 1930, Minshu Vol. 9, p. 1049; Judgement of the Great Court of Judicature, 1 March 1938, Minshu Vol. 17, p. 318; Judgement of the Great Court of Judicature, 26 November 1940, Minshu Vol. 19, p. 2088; Judgement of Supreme Court, 22 February 1957, Minshu Vol. 11, No. 2, p. 350; Wagatsuma (n. 9), p. 341; Nakai (n. 7), p. 396; Oho (n.9), p. 417; Okuda (n. 9), p. 574; Ishida (n. 9), p. 335; Shiomi (n. 8), p. 283; Nakata (n. 3), pp. 473 f.

^23^
Judgement of the Great Court of Judicature, 13 November 1914, Minroku Vol. 20, p. 922; Judgement of the Great Court of Judicature, 24 October 1930, Minshu Vol. 9, p. 1049; Judgement of Supreme Court, 22 February 1957, Minshu Vol. 11, No. 2, p. 350.

^24^
Judgement of the Great Court of Judicature, 26 November 1940, Minshu Vol. 19, p. 2088.

^25^
Judgement of the Great Court of Judicature, 1 March 1938; Minshu Vol. 17, p. 318.

^26^
Nakai (n. 7), p. 397; Shiomi (n. 8), p. 285; Nakata (n. 3), p. 474.

^27^
Judgement of Supreme Court, 4 March 1976, Minshu Vol. 30, No. 2, p. 48; Judgement of Supreme Court, 21 September 1978, Hanji No. 907, p. 54.

^28^
Wagatsuma (n. 9), p. 330; Okuda (n. 9), pp. 575 f.; Ishida (n. 9), p. 338; Nakata (n. 3), p. 473.

^29^
Nakai (n. 7), p. 398.

^30^
Nakata (n. 3), p. 473.

^31^
Justice Ministry’s Legislative Council Civil Code (Obligation Law) Subcommittee Document 80-3, p. 29; Nakata (n. 3), p. 475.

^32^
Justice Ministry’s Legislative Council Civil Code (Obligation Law) Subcommittee Document 80-3, p. 29; Takeo Tsutsui and Hideki Muramatsu (eds.), *A Question-and-Answer Civil Code (Obligation Law) Revision*, (Tokyo: Shoji Houmu, 2018), p. 202.

^33^
Shiomi (n. 8), p. 290; Nakata (n. 3), p. 475. Hiroyuki Hirano insists that it should be interpreted widely as normal intention. See Hirano (n. 10), pp. 523 f.

^34^
Justice Ministry’s Legislative Council Civil Code (Obligation Law) Subcommittee Document 83-2, p. 33; Nakata (n. 3), p. 475.

^35^
Yuka Fukagawa, ‘Set-Off (2) - Prohibition of Set-Off’ in Yoshio Shiomi et al. (eds.), *The Civil Code Revision Answer Book*, (Tokyo: Shoji Houmu, 2018), p. 363.

^36^
Shiomi (n. 8), pp. 290 f.

^37^
Justice Ministry’s Legislative Council Civil Code (Obligation Law) Subcommittee Document 80-3, p. 29.

^38^
Nakata (n. 3), p. 476.

^39^
Justice Ministry’s Legislative Council Civil Code (Obligation Law) Subcommittee Document 83-2, p. 33; Tsutsui and Muramatsu (n. 32), p. 202; Nakata (n. 3), p. 476.

^40^
Nakata (n. 3), p. 477.

^41^
Tsutsui and Muramatsu (n. 32), p. 203; Nakata (n. 3), p. 477.

^42^
Art. 152 of the Civil Enforcement Act; Art 11 (3) of the Pension Act; Art. 24 of the National Pension Act; Art. 41 (1) of the Employees’ Pension Insurance Act; Art. 58 of the Public Assistance Act; Art. 15 of the Child Allowance Act; Art. 83 (2) of the Labor Standards Act, etc.

^43^
Wagatsuma (n. 9), p. 331; Yoshio Nakai, ‘Article 510 Civil Code’ in Tetsu Isomura (ed.), *Commentary of the Civil Code (12) Obligation (3)* (Tokyo: Yuhikaku, 1970), p. 435; Shiomi (n. 8), pp. 293 f.; Nakata (n. 3), p. 478; Hirano (n. 10), p. 525.

^44^
Tsutsui and Muramatsu (n. 32), p. 204. See also BGB Art. 392, FCC Art. 1347-7 and Art. 1348-1 (3).

^45^
Nakata (n. 3), pp. 484 ff.

^46^
Nakata (n. 3), pp. 486 f.

^47^
Shiomi (n. 8), p. 281; Yasuto Nishiuchi, ‘Article 676 Civil Code’ in Yutaka Yamamoto (ed.), *New Commentary of the Civil Code (14) Obligation (7)* (Tokyo: Yuhikaku, 2018), p. 575; Yoshio Shiomi, *New Special Theory of Contract Law II* (Tokyo: Shinzansha, 2021), p. 450; Hiroyasu Nakata, *Contract Law* (new ed., Tokyo: Yuhikaku, 2021), p. 584.

^48^
Nishiuchi (n. 47), p. 571; Shiomi (n. 47), p. 442.

^49^
Makoto Arai, *Trust Law* (4th ed., Tokyo: Yuhikaku, 2014), p. 357; Kiyoe Kado, ‘Article 22 Trust Act’, in Hiroto Dougauchi (ed.), *Commentary of Trust Act* (Tokyo: Kobundo, 2017), p. 107.

^50^
Wataru Tanaka, *Companies Law* (3rd ed., Tokyo: The University of Tokyo Press, 2021), p. 506, Kenjiro Egashira, *Law of Company Limited* (8th ed., Tokyo: Yuhikaku, 2021), p. 38 and p. 780.

^51^
Masaru Noma, ‘Article 17 Labor Standards Act’ in Souya Aoki and Noboru Kataoka (eds.), *Labor Standards Act I* (Tokyo: Seirin Shoin, 1994), pp. 228 f.; Hisaaki Fujikawa, ‘Article 17 Labor Standards Act’ in University of Tokyo Collegium Labor Law, *Commentary of the Labor Standards Law vol. 1* (Tokyo: Yuhikaku, 2003), pp. 294 f.

^52^
Makoto Ito, et al., *Commentary of the Bankruptcy Act* (3rd ed., Tokyo: Kobundo, 2020), p. 548.

^53^
Kanichiro Douzono, ‘Article 103 Bankruptcy Act’ in Morio Takeshita (ed.), *Great Commentary of the Bankruptcy Act* (Tokyo: Seirin Shoin, 2007), p. 431; Ito, et al. (n. 52), p. 783.

^54^
Katsumi Yamamoto, ‘Article 67 Bankruptcy Act’ in Morio Takeshita (ed.), *Great Commentary of the Bankruptcy Act* (Tokyo: Seirin Shoin, 2007), p. 292; Nobuhiro Oka, ‘Article 67 Bankruptcy Act’ in Mutsuo Tanaka and Kazuhiko Yamamoto (eds), *Commentary of the Bankruptcy Act vol. 1* (Kinzai Institute for Financial Affairs, 2015), pp. 471 f.; Ito, et al. (n. 52), p. 554; Makoto Ito, *Bankruptcy Act and Civil Rehabilitation Act* (5th ed., Tokyo: Yuhikaku, 2022), p. 521.

^55^
Ito, et al. (n. 52), p. 563.

^56^
Katsumi Yamamoto, ‘Article 69 Bankruptcy Act’ in Morio Takeshita (ed.), *Great Commentary of the Bankruptcy Act* (Tokyo: Seirin Shoin, 2007), p. 302; Nobuhiro Oka, ‘Article 69 Bankruptcy Act’ in Mutsuo Tanaka and Kazuhiko Yamamoto (eds.), *Commentary of the Bankruptcy Act vol. 1* (Kinzai Institute for Financial Affairs, 2015), p. 483; Ito, et al. (n. 52), p. 561; Ito (n. 54), p. 522.

^57^
Katsumi Yamamoto, ‘Right to Set-Off’ in Teiichiro Nakano and Toru Michishita (eds.), *Basic Commentary of the Bankruptcy Act* (2nd ed., Tokyo: Nippon Hyoronsha, 1997), p. 158; Yamamoto (n. 54), p. 294.

^58^
Kazuhiko Yamamoto, ‘Lease Contract’ in National Network of Lawyers for Insolvency Resolution (ed.), *Commentary on Issues of New Bankruptcy Act vol. 1* (Kinzai Institute for Financial Affairs, 2005), p. 100 and p. 104 (note 46); Masami Okino, ‘Right to Set-Off’ in Kazuhiko Yamamoto et al., *Outline of Insolvency Law* (supplementary 2nd ed., Tokyo: Kobundo, 2015), p. 253; Ito (n. 54), pp. 524 f.

^59^
Ito, et al. (n. 52), pp. 556 f. See also Judgement of Supreme Court, 17 January 2005, Minshu Vol. 59, No. 1, p. 1.

^60^
Yamamoto (n. 54), p. 293; Oka (n. 54), p. 474; Ito, et al. (n. 52), p. 556.

^61^
Katsumi Yamamoto, ‘Article 71 Bankruptcy Act’ in Morio Takeshita (ed.), *Great Commentary of the Bankruptcy Act* (Tokyo: Seirin Shoin, 2007), p. 305; Eiichi Obata, ‘Article 71 Bankruptcy Act’ in Mutsuo Tanaka and Kazuhiko Yamamoto (eds), *Commentary of the Bankruptcy Act vol. 1* (Kinzai Institute for Financial Affairs, 2015), pp. 488 f.; Ito, et al. (n. 52), p. 567.

^62^
Yamamoto (n. 61), p. 306; Obata (n. 61), p. 491; Ito, et al. (n. 52), p. 570.

^63^
Judgement of the Great Court of Judicature, 23 December 1926, Shimbun No. 2660, p. 15 (related to the old law).

^64^
Judgement of the Great Court of Judicature, 31 July 1936, Minshu Vol. 15, p. 1563; Judgement of Supreme Court, 24 March 1964, Hanji No. 370, p. 30 (both related to the old law).

^65^
Judgement of Supreme Court, 17 January 2005, Minshu Vol. 59, No. 1, p. 1 (related to the old law).

^66^
Yamamoto (n. 61), p. 307; Obata (n. 61), p. 493; Ito, et al. (n. 52), p. 570.

^67^
Yamamoto (n. 61), p. 308; Obata (n. 61), pp. 493 ff.; Ito, et al. (n. 52), pp. 574 f.

^68^
Yamamoto (n. 61), p. 309; Obata (n. 61), p. 495; Ito, et al. (n. 52), p. 575.

^69^
Yamamoto (n. 61), p. 309.

^70^
Yamamoto (n. 61), p. 309; Obata (n. 61), p. 495; Ito, et al. (n. 52), p. 576.

^71^
Ito, et al. (n. 52), p. 576.

^72^
Judgement of Supreme Court, 28 May 2012, Minshu Vol. 66, No. 7, p. 3123.

^73^
Judgement of Supreme Court, 28 May 2012, Minshu Vol. 66, No. 7, p. 3123.

^74^
Ito, et al. (n. 52), p. 582.

^75^
Yamamoto (n. 61), p. 309 and Katsumi Yamamoto, ‘Article 72 Bankruptcy Act’ in Morio Takeshita (ed.), *Great Commentary of the Bankruptcy Act* (Tokyo: Seirin Shoin, 2007), p. 314; Obata (n. 61), pp. 495 f.; Ito, et al. (n. 52), p. 576.

^76^
Yamamoto (n. 61), p. 310; Ito, et al. (n. 52), p. 577.

^77^
Obata (n. 61), pp. 496 ff.; Ito, et al. (n. 52), pp. 577 ff.

^78^
Judgement of Supreme Court, 18 October 1988, Minshu Vol. 42, No. 8, p. 575 (related to the old law).

^79^
Judgement of Supreme Court, 2 November 1965, Minshu Vol. 19, No. 8, p. 1927 (related to the old law); Judgement of Supreme Court, 14 April 1998, Minshu Vol. 52, No. 3, p. 813 (composition case).

^80^
Yamamoto (n. 61), p. 310; Obata (n. 61), p. 497; Ito, et al. (n. 52), p. 578. See also Judgement of Supreme Court, 26 February 1985, Kinhou No. 1094, p. 38 (related to the old law).

^81^
Judgement of Supreme Court, 5 June 2014, Minshu Vol. 68, No. 5, p. 462 (rehabilitation case).

^82^
Judgement of Supreme Court, 8 September 2020, Minshu, Vol. 74, No. 6, p. 1643.

^83^
Yamamoto (n. 61), p. 310; Obata (n. 61), p. 498; Ito, et al. (n. 52), p. 581.

^84^
Yamamoto (n. 75), pp. 315 f.; Eiichi Obata, ‘Article 72 Bankruptcy Act’ in Mutsuo Tanaka and Kazuhiko Yamamoto (eds), *Commentary of the Bankruptcy Act vol. 1* (Kinzai Institute for Financial Affairs, 2015), p. 504; Ito, et al. (n. 52), p. 586.

^85^
Yamamoto (n. 75), p. 316; Ito, et al. (n. 52), p. 586.

^86^
See 1-1-2 and 2-5-1.

^87^
See 1-1-2 and 2-5-2.

^88^
See 3-2-1 and 3-2-2.

^89^
See 3-4.
