# Supplementary material for: Requirements for set-off in Japanese law
Source: F1000Res. 2025 Jun 3;14:255. Originally published 2025 Mar 3. [Version 2] doi: 10.12688/f1000research.161862.2 (PMC12335724; doi:10.12688/f1000research.161862.2)
Supplement: Peer review history

# Reviewer report: Reviewer response for version 2

**DOI:** 10.5256/f1000research.183198.r394783

**Published:** 2025-08-22

**Authors:** Charles Ho Wang Mak

**Affiliations:** 1 University of Bristol School of Law, Bristol, England, UK

This article offers a valuable and much-needed account of Japanese set-off law in English. It provides a comprehensive overview of the requirements for set-off under Japanese law, a topic that has not been extensively discussed in international literature. In particular, the article's systematic breakdown of set-off into affirmative and negative requirements is excellent. Your use of specific examples and clear explanations of concepts like "Sousai-Tekijou" (eligibility for set-off) and the distinction between active and passive claims makes the complex topic approachable. The section on set-off in bankruptcy proceedings is also particularly strong. 

 However, you should consider a few key improvements:

1. While the abstract provides a good overview, a formal introduction would set the stage, state the paper's aim, and highlight its contribution to the English-language literature.
2. Your response acknowledges that some passages are difficult to translate directly from Japanese legal texts. I suggest a collaborative effort with a native English speaker with a legal background to smooth out these parts. The goal is not to change the meaning but to improve readability for an international audience. Specific sections that could benefit from this include the third paragraph under 1-1-2 and the first paragraph under 3-4-2.
3. While you intentionally avoided a full comparative study for the sake of conciseness, a brief, well-structured section in the main body (not just the conclusion) could address the German and French law comparisons. This could also be an opportunity to clarify or revise statements, such as the one about German law's stance on knowledge of assignment (BGB Art. 392 and 406).
4. If possible, the complexity of set-off, especially in a tripartite relationship or in bankruptcy, would be significantly easier to understand with a few simple diagrams. These visuals could illustrate the flow of claims and the impact of concepts like attachment and assignment.

Is the review written in accessible language?

Partly

Are all factual statements correct and adequately supported by citations?

Yes

Are the conclusions drawn appropriate in the context of the current research literature?

Yes

Is the topic of the review discussed comprehensively in the context of the current literature?

Partly

Reviewer Expertise:

Insolvency law, sovereign debt restructuring, trust law, financial regulation, technology law, and dispute resolution

I confirm that I have read this submission and believe that I have an appropriate level of expertise to confirm that it is of an acceptable scientific standard, however I have significant reservations, as outlined above.

# Reviewer report: Reviewer response for version 2

**DOI:** 10.5256/f1000research.183198.r389550

**Published:** 2025-08-12

**Authors:** Moritz Bälz

**Affiliations:** 1 Goethe University Frankfurt, Frankfurt Am Main, Germany

In my view, the revision has improved the article. In particular, it was reasonable to clarify the article's aim and to avoid raising expectations for a comparative analysis. 

 I have no further comments.

Is the review written in accessible language?

Partly

Are all factual statements correct and adequately supported by citations?

Yes

Are the conclusions drawn appropriate in the context of the current research literature?

Yes

Is the topic of the review discussed comprehensively in the context of the current literature?

Partly

Reviewer Expertise:

Japanese business law from a comparative perspecitve. However, I am not an insolvency expert.

I confirm that I have read this submission and believe that I have an appropriate level of expertise to confirm that it is of an acceptable scientific standard.

# Reviewer report: Reviewer response for version 2

**DOI:** 10.5256/f1000research.183198.r394786

**Published:** 2025-08-09

**Authors:** Rebecca Parry

**Affiliations:** 1 Nottingham Trent University, Nottingham, UK

This article provides a systematic doctrinal discussion of set-off under Japanese law.  Since this is an area of law that attracts limited discussion internationally this will be an important account, covering key statutes and case law.  It will be helpful for anyone who is aiming to understand the operation of set-off both in insolvency and outside of it in Japan. 

 In terms of readability, an introduction should be added to explain to the reader the aim of the paper.  This can be used to highlight the important laws and what has influenced their development.  Also to highlight the contribution that this article makes to the literature.   I believe that it is the only recent source that will discuss set-off under Japanese law in English.  

 The article includes a good number of references.  It is good practice to indicate where texts are in the Japanese language and to give the Japanese titles.

 The material would have been enhanced with a greater comparative element but is still valuable for the reasons already given.

Is the review written in accessible language?

Yes

Are all factual statements correct and adequately supported by citations?

Yes

Are the conclusions drawn appropriate in the context of the current research literature?

Yes

Is the topic of the review discussed comprehensively in the context of the current literature?

Yes

Reviewer Expertise:

International and comparative insolvency law, company law, law and technology

I confirm that I have read this submission and believe that I have an appropriate level of expertise to confirm that it is of an acceptable scientific standard.

# Reviewer report: Reviewer response for version 1

**DOI:** 10.5256/f1000research.177953.r378392

**Published:** 2025-05-02

**Authors:** Moritz Bälz

**Affiliations:** 1 Goethe University Frankfurt, Frankfurt Am Main, Germany

This article concisely sets out the requirements for set-off under Japanese law, based on relevant case law and selected academic opinions. The author places particular emphasis on set-off in the case of insolvency. I am not aware of any comparable account in English. Therefore, this article represents a welcome addition to the literature and, once slightly revised, should also serve as a useful initial orientation for legal practitioners.

 The rules of set-off under Japanese law distinguish between (1) positive requirements that must be met for set-off, and (2) negative requirements that may result in the debtor's right of set-off being excluded even if the positive requirements are met. The rules of set-off are (3) modified in the Bankruptcy Act for the case of insolvency. The structure of the article at hand is also based on this three-part structure, making it clear and logical.

 The Abstract emphasizes at the beginning that the rules of set-off in Japanese law were created at the end of the 19th century based on foreign, in particular German, models, but have since been developed independently by the Japanese courts and revised by the legislator of the 2017 reform of the law of obligations. Looking at the abstract, it is surprising that the article discusses similarities and differences between current Japanese law and foreign legal systems, such as German law, only at the very end of the article in the Conclusion, and even there only briefly. In my view, the article would benefit if these comparative considerations were discussed in greater depth. If this turns out to be too cumbersome – the devil is in the details -, one might opt to shorten the reference to the genesis of current Japanese rules at the beginning of the Abstract to avoid raising false expectations.

 The actual article begins somewhat abruptly with the positive requirements for set-off (1). It would be more reader-friendly to begin with a brief introduction that could, for example, emphasize the functions of set-off. The reciprocity of the claims is discussed as the first positive requirement. The decision of the Supreme Court of July 8, 2016, which the author cites as an illustration, is presented somewhat tersely at this point. One might add that the requirement of reciprocity under Art. 505 (1) Japanese Civil Code and Art. 92 (1) Japanese Civil Rehabilitation Act are similar in this respect. As a second positive condition, the author discusses the existence of both claims. In this respect, an important exception exists with regard to time-barred claims, which, while extinguished, can still be set off under certain conditions. The third positive requirement is the similarity of the claims with regard to their content. Remarkably, such similarity is assumed to exist even for monetary claims in different currencies. Finally, both claims must be due in principle, whereby the party making the set-off can fulfill its own claim prematurely.

 Even if the positive conditions for offsetting are met, a set-off may be excluded under special circumstances (2). The author systematically presents these so-called negative requirements, explains their purpose, and briefly illustrates the various scenarios using examples from case law. There are certain claims which, by their nature, cannot be used for set-off, such as a right that another person refrain from doing something. Furthermore, set-off is generally excluded with a claim that is subject to a defense (the author points out an important exception recognized by the Supreme Court in the case of work contracts). A set-off can be contractually excluded, however, such an exclusion can only be asserted against third parties if they were either aware of the exclusion or were unaware of it due to gross negligence. It is not possible to make a set-off against certain claims for damages in tort or damages for the death or injury of a person. These latter rules are intended to ensure that the victims receive actual payment. If the passive claim has been attached, generally a set-off can no longer be made against it (again, some exceptions apply). Finally, a set-off may be excluded by special statutory provisions.

 In the third part (3), the author specifically addresses set-off in the context of insolvency proceedings. Since I am not a true insolvency expert, I can assess this part only to a limited extent. However, the Overview at the beginning of the section seems to me to nicely illustrate the special interests involved in insolvency. The security function of set-off is particularly important where the other party has become insolvent. The Japanese Bankruptcy Code takes this into account by lowering the requirements for a set-off compared to the general rules of civil law, both for the claim to be set-off as well as the claim against which the set-off is to be made (e.g., the requirements of similarity and maturity of the claims). On the other hand, the Bankruptcy Act also restricts the setting off of insolvency claims in some respects in the interest of equal treatment of all creditors. Certain statutory prohibitions on set-off do not apply in the case of insolvency, as it is considered important to protect a creditor who has relied on the possibility of realizing his claim by way of set-off.

 In his Conclusion, the author emphasizes that Japanese law provides particularly extensive protection for reliance on the possibility of set-off. At this point, the author returns to the German origins of the Japanese rules and cites two scenarios where German law seems more restrictive. The first example, I believe, does not quite accurately reflect the content of German law (BGB Art. 392 and 406 refer to the knowledge of the assignment, not the assignment as such). The author's overall conclusion is nevertheless plausible as a general trend.

 The article is largely readable, with only a few passages becoming difficult to understand (third paragraph under 1-1-2, first sentence under 3-3-1-1, second paragraph under 3-4-1, and first paragraph under 3.4.2). A linguistic revision by a native speaker would help smooth out some of the wording. Some books cited are not cited in the latest edition (e.g., Egashira, Nakata, Tanaka). The translation of the Japanese book titles sometimes sounds a bit strange (“Law of Company Limited”, “General Theory of Obligation Law”, etc.).

 I hope my comments can be helpful.

Is the review written in accessible language?

Partly

Are all factual statements correct and adequately supported by citations?

Yes

Are the conclusions drawn appropriate in the context of the current research literature?

Yes

Is the topic of the review discussed comprehensively in the context of the current literature?

Partly

Reviewer Expertise:

Japanese business law from a comparative perspecitve. However, I am not an insolvency expert.

I confirm that I have read this submission and believe that I have an appropriate level of expertise to confirm that it is of an acceptable scientific standard, however I have significant reservations, as outlined above.

# Response

**Published:** 2025-05-05

**Authors:** Hiroki OKAMOTO

**Affiliations:** Not Applicable, Japan

I sincerely appreciate your peer review. As you kindly understand, this article as review aims to provide a concise introduction to the requirements for set-off under current Japanese law and the general understanding regarding them. For this purpose, the review is devoted to describing objectively the current state of Japanese set-off law without interjecting my humble opinion.

 And I fully agree that comparative study holds high academic and practical significance. However, for comparative research, detailed explanations of other countries' legal systems are indispensable. For example, in order to compare Japanese law with German law, it is essential to address not only the set-off law itself but also the institutional differences regarding assignment of claims and attachment. These explanations may undermine the conciseness that this article prioritizes. Therefore, descriptions related to German and French law are kept to a minimum.

 Nonetheless, as you indicate, the current version of the text may inadvertently set readers’ expectations about comparative research. Other reviewers have also pointed out the inadequacy of the comparative study. Therefore, I would like to revise the text in accordance with your advice.

 Additionally, I will strive to rewrite the passages that are deemed difficult to understand. However, since they are based on the wording of Japanese legal texts and extremely difficult to translate into English, so I am unsure how much they can be refined. In particular, the passage concerning the assignment of claims is founded on the distinctive framework of Japanese law. Furthermore, this paper has been reviewed by a native speaker.

# Reviewer report: Reviewer response for version 1

**DOI:** 10.5256/f1000research.177953.r378393

**Published:** 2025-04-24

**Authors:** Matthias Lehmann, Emeric Prévost

**Affiliations:** 1 University of Vienna, Vienna, Austria; 2 Research Fellow, University of Vienna, Vienna, Austria

Review of Hiroki Okamoto, “Requirements for set-off in Japanese law”

 By Matthias Lehmann and Emeric Prévost

 This article offers a detailed presentation of the Japanese law of set-off currently in force. It also includes useful considerations on the functioning of set-off rules during bankruptcy proceedings. The different rules contained in the Japanese Civil Code and the Japanese Bankruptcy Act are carefully outlined by the author, with stylized examples to facilitate the understanding of their functioning and purpose. Helpful references are also made to authoritative analyses and case law. This said, visuals such as diagrams are lacking; these would have been particularly helpful to explain the complex functioning and interplay of rules, and to avoid the confusion that merely abstract descriptions may create.

 The rules carefully examined by the author are the result of a reform of the Japanese Civil Code in 2017 (Act No. 44 of 2017 for the Partial Revision of the Civil Code) that notably aimed to modernise the law of obligations. Although the author often points out the purpose of each commented provision, there is no critical and systematic analysis of whether current rules represent a real and significant improvement compared to the previous state of the law. Such a diachronic analysis would have been particularly useful to the reader to better understand the underlying principles of the current Japanese rules on set-off.

 The paper interestingly engages in some comparative law analysis, especially with some German law provisions. However, one may regret that the author does so only selectively and in a rather summary fashion. Adding to that, the few comparative observations of the paper only appear in the conclusion. One may thus be quite surprised by the fact that the author starts his concluding remarks by stating that “as previously explained, the basic requirements for set-off in Japanese law have many things in common with other civil law countries”. Although French law, for instance, is mentioned in the abstract, the paper does not actually contain any other reference to the French legal system. In sum, it would be worth undertaking a more consistent comparative study. 

 A proper comparative analysis would indeed allow for a better understanding of the comparative advantages or disadvantages of Japanese law in the broader context of the world’s legal systems. The insights gained from such a critical study would be particularly valuable not only for scholars but also for the legal practice, as transnational businesses are widely recognised across jurisdictions the freedom to choose the most suitable law to their contractual relationships.

 Overall, the paper provides a detailed and interesting overview of the Japanese rules on set-off. One can hope, however, that this is only the first step of a more critical and comparative study.     

Is the review written in accessible language?

Partly

Are all factual statements correct and adequately supported by citations?

Yes

Are the conclusions drawn appropriate in the context of the current research literature?

Partly

Is the topic of the review discussed comprehensively in the context of the current literature?

Yes

Reviewer Expertise:

private law, comparative law, private international law

We confirm that we have read this submission and believe that we have an appropriate level of expertise to confirm that it is of an acceptable scientific standard, however we have significant reservations, as outlined above.

# Response

**Published:** 2025-04-26

**Authors:** Hiroki OKAMOTO

**Affiliations:** Not Applicable, Japan

I sincerely appreciate your peer review. The feedback and comments provided are generally understandable and ratiocinative. However, I am concerned that you have a different understanding of the article type “review” from my view.

 This paper is written not as a research article, but as a review. This review aims to provide a concise introduction to the requirements for set-off under current Japanese law and the general understanding regarding them. The intended audience includes lawyers and business people who cannot read Japanese.

 For this purpose, the review is devoted to describing objectively the current state of Japanese set-off law without interjecting my humble opinion. Consequently, critical study based on subjective evaluation is not included.

 Additionally, conciseness has priority in this paper. Therefore, to avoid elaborating on peripheral information, it does not include explanations of the differences from the pre-revision law, the purpose of the revision, or comparative studies.

 Especially when conducting comparative research, detailed explanations of other countries' legal systems are indispensable. For example, in order to compare Japanese law with German law, it is essential to address not only the set-off law itself but also the institutional differences regarding assignment of claims and attachment. Additionally, when comparing with French law, it is necessary to analyze unique French concepts such as judicial set-off and the interconnection of obligations. However, these explanations may not necessarily be beneficial to the intended audience who seeks information on Japanese law. Therefore, descriptions related to German and French law are kept to a minimum.

 Taking the above into consideration, this paper has been published as a review that concisely summarizes a part of of Japanese set-off law. I would greatly appreciate it if you could understand the intent of the review. Finally, I fully agree that a more critical and comparative study holds higher academic and practical significance.
